# Advancements in Pediatric Audiological Assessments Using Wideband Acoustic Immittance: A Review

**DOI:** 10.3390/audiolres14040058

**Published:** 2024-08-14

**Authors:** Wen Jiang, Yi Mu, Fei Zhao, Peng Wang

**Affiliations:** 1 Department of Otolaryngology, The Affiliated Hospital of Xuzhou Medical University, Xuzhou 221000, China; wen.jiang@xzhmu.edu.cn; 2 The College of Medical Technology, Xuzhou Medical University, Xuzhou 221000, China; 302102110204@stu.xzhmu.edu.cn; 3 The Second College of Clinical Medicine, Xuzhou Medical University, Xuzhou 221000, China; 4 Auditory Engineering Laboratory of Jiangsu Province, Xuzhou 221000, China; 5 Centre for SLT and Hearing Sciences, Cardiff School of Sport and Health Sciences, Cardiff Metropolitan University, Cardiff CF5 2YB, UK; fzhao@cardiffmet.ac.uk; 6 National Intellectual Property Information Service Center, China University of Mining and Technology, Xuzhou 221000, China

**Keywords:** pediatric audiology, wideband acoustic immittance, audiological assessments

## Abstract

Objectives: This study’s objectives were to explore the potential of wideband acoustic immittance (WAI) as a diagnostic tool, examining its accuracy and efficiency in pediatric audiology. Methods: A narrative review of the contemporary literature was conducted, focusing on studies that assessed the use of WAI in diagnosing pediatric auditory conditions. Key variables such as diagnostic accuracy, efficiency, and clinical outcomes were considered. Results: This review highlighted that WAI offers a broader range of test frequencies and more comprehensive diagnostic information compared with traditional tympanometry. The studies indicated that WAI has the potential to improve diagnostic accuracy and efficiency in pediatric audiology. Distinct patterns of wideband absorbance were identified, enabling more detailed and accurate diagnostic evaluations. Conclusions: WAI shows substantial potential as a diagnostic tool in pediatric audiology, offering improvements in diagnostic accuracy and efficiency over traditional methods. While the initial findings are promising, further research is needed to fully understand its applicability and benefits across different pediatric populations. Future studies should aim to validate the clinical utility of WAI to ensure its widespread adoption in pediatric audiological assessments.

## 1. Introduction

Acoustic immittance testing is fundamental to pediatric audiology, encompassing both acoustic impedance and its reciprocal, acoustic admittance [1]. Acoustic admittance measures the ease with which acoustic energy passes through a system, consisting of conductance (the real part) and susceptance (the imaginary part) [1]. In contrast, acoustic impedance quantifies the resistance a system offers to acoustic energy flow due to applied acoustic pressure, expressed as resistance (the real part) and reactance (the imaginary part) [1]. Traditional acoustic immittance tests include single-frequency (e.g., 226 Hz) tympanometry, acoustic reflexes, and acoustic reflex decay.

Recently, wideband acoustic immittance (WAI) has shown significant promise in enhancing existing clinical techniques. WAI is a comprehensive audiological assessment tool that measures the energy reflectance and absorbance in the ear canal across a wide frequency range, providing detailed information about middle-ear function [2]. Energy reflectance refers to the proportion of sound energy that is reflected back from the middle ear when sound waves enter the ear canal [2], providing insights into the effectiveness of sound transmission to the inner ear. Energy absorbance, meanwhile, indicates the amount of sound energy absorbed by the middle-ear structures [3]. These parameters are derived from the same underlying principles of acoustic immittance, where high reflectance suggests greater impedance and low absorbance, whereas high absorbance suggests lower impedance and higher admittance.

This review examines WAI’s role in pediatric hearing assessments, focusing on its technical aspects and clinical uses. Studies have mentioned that acoustic immittance can be a sensitive method to detect changes in middle- and inner-ear functions [2,4]. However, traditional acoustic immittance (e.g., 226 Hz) uses a single frequency and is less effective in diagnosing certain ear diseases, increasing the risk of misdiagnosis [2]. Improving diagnostic efficiency is a significant challenge in this field. WAI provides a broader range of test frequencies and more comprehensive diagnostic information than traditional clinical tympanometry, which typically uses a single frequency (e.g., 226 Hz) and may not detect certain ear diseases [3]. Additionally, WAI can complement other clinical tests, such as pure-tone audiometry, by offering detailed insights into middle-ear function. For instance, patients with similar audiograms showing conductive hearing loss might exhibit entirely different WAI results. One patient might present with middle-ear conductive hearing loss due to abnormal middle-ear function, while another might have inner-ear conductive hearing loss resulting from changes in inner-ear pressure. In such cases, it is crucial to cross-validate pure-tone audiometry results with WAI findings to accurately diagnose the underlying issue. WAI uses frequencies from 226 to 8000 Hz, making it more sensitive to changes in both mass and stiffness components and for detecting minor changes in transmission characteristics [2,4,5,6]. The objective of this review is to clarify the operational principles of WAI and to assess its clinical utility in diagnosing pediatric auditory disorders. Through an analysis of the existing literature, this review aims to evaluate WAI’s diagnostic capabilities in comparison with traditional methods and to identify areas for future research. This exploration aims to contribute to the understanding of WAI’s application in pediatric audiology, highlighting its potential impact on clinical practice and research within this field.

## 2. Establishing Normative Data for Pediatric Wideband Acoustic Immittance

### 2.1. Comparative Analysis of Wideband Absorbance at Different Ages

Throughout early childhood, the auditory system undergoes significant anatomical and functional transformations. In neonates, the external auditory canal predominantly comprises cartilaginous structures, transitioning to ossification from the fourth month [7]. Concurrently, the ossicular chain’s bone density and interconnectivity incrementally increase, leading to notable alterations in the acoustic properties of the middle ear [7].

Recent studies [8,9] have advanced our understanding of pediatric auditory function, particularly through the use of WAI technology. Tympanometry is a clinical test that measures the movement of the eardrum in response to changes in air pressure, typically using a low-frequency (e.g., 226 Hz) probe tone for individuals older than 6 months and a higher-frequency (e.g., 1000 Hz) probe tone for those younger than 6 months [10]. It is a standard practice in audiology to assess middle-ear function [11]. It increases sensitivity to acoustic impedance at 1000 Hz in infants aged 4 to 6 months compared with the traditionally used 226 Hz, indicating a developmental shift in auditory properties [8]. Sanford and his colleagues demonstrated the diagnostic advantages of WAI over standard 1000 Hz tympanometry in neonatal middle-ear functional analysis [9].

**Figure 1:**
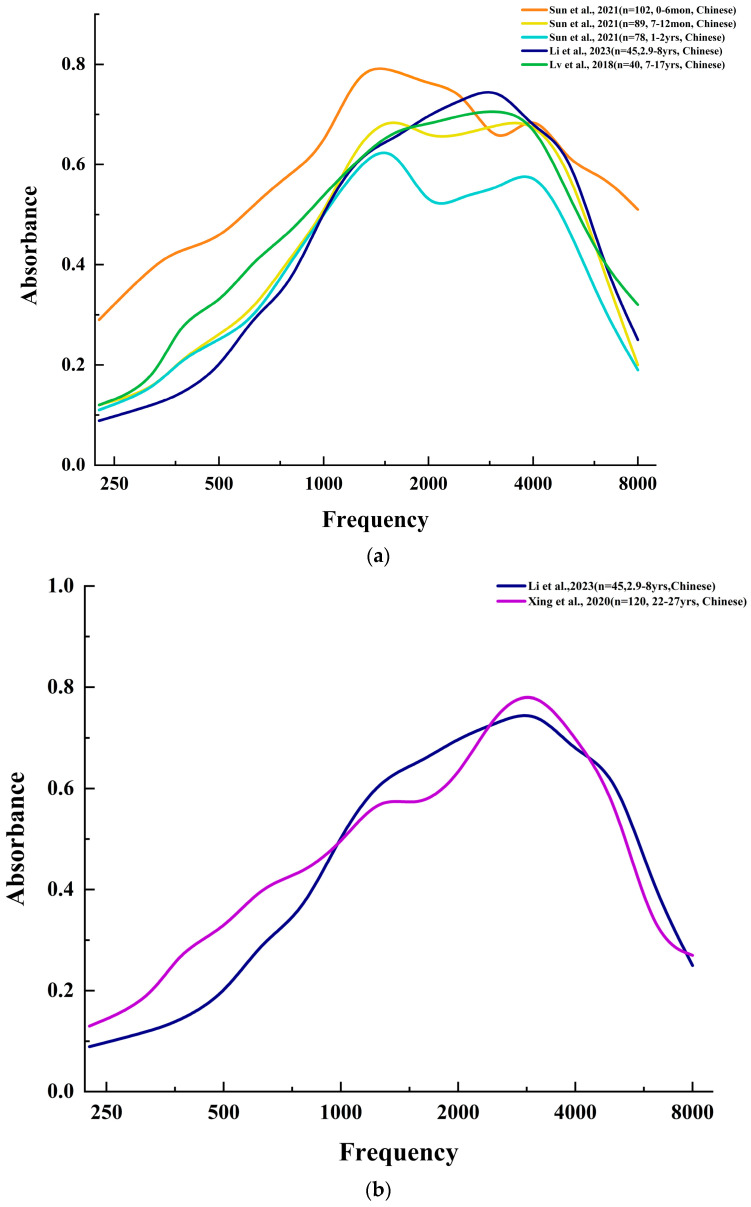

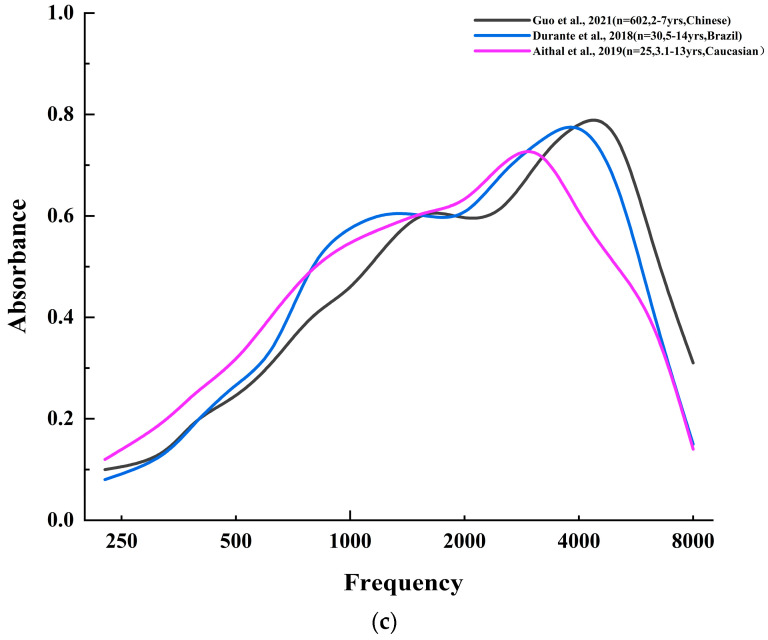
Establishment of normal WAI values for children (**a**) at different ages [21,22,23], (**b**) compared with adults [21,24], and (**c**) of different races [4,18,19].

Wideband absorbance (WBA) is the main component in WAI measurement and has been extensively studied [12,13,14]. Lin et al. [12] identified significant differences in WBA between groups that passed and failed hearing screenings, suggesting WBA’s potential as a reliable indicator. Peng et al. [13] explored WBA patterns in infants aged 1 to 5 months, and Stuppert et al. [14] expanded the age range of their study, categorizing children into four distinct age groups. These studies revealed significant variations in frequency–WBA profiles across age categories, indicating ongoing auditory system development with distinct stages marked by different acoustic characteristics. Together, these findings highlight the critical role of age-specific assessments in pediatric audiology (Figure 1a).

### 2.2. Comparative Analysis of WBA Patterns in Both Children and Adults

Further insights into developmental changes were provided by Pan and Yang [15], who conducted a comparative analysis of WBA patterns in both children and adults. Their study found broadly similar WBA trends across age groups but significant discrepancies at specific frequencies. Beers et al. [16] observed elevated WBA in children compared with adults, particularly within 1250–2500 Hz. These frequency discrepancies are significant as they indicate different mechanisms of sound energy transmission from the middle ear to the inner ear in children compared with adults. This difference in response can be attributed to factors like the anatomical and physiological development of the auditory system, which significantly varies between children and adults. This suggests that pediatric and adult auditory systems process sound differently, highlighting the need for age-specific assessment criteria rather than applying adult standards to children [11]. Such insights are crucial for improving the accuracy of pediatric audiological diagnostic approaches, ensuring that assessments and treatments are appropriately tailored to the developmental stage of the child’s auditory system. Shahnaz et al. [17] found distinct WBA curve patterns between adults and children, with adults generally exhibiting a bimodal curve and children exhibiting a unimodal curve, highlighting developmental aspects of auditory function (Figure 1b). These studies emphasize the importance of considering age as a critical factor in audiological assessments and interventions.

### 2.3. Comparative Analysis of Wideband Absorbance between Ethnicities

Moreover, WBA results significantly vary across different ethnic groups. Studies comparing Chinese [18], Brazilian [19], and Caucasian [4] children showed that all groups exhibit a general trend of increasing absorbance with frequency, peaking between 2000 Hz and 4000 Hz before declining. Notably, Chinese children display a higher peak at around 4000 Hz, Brazilian children have a broad peak slightly above 2000 Hz, and Caucasian children peak between 2000 Hz and 4000 Hz. These differences highlight the need for ethnicity-specific normative data in pediatric audiology to ensure accurate diagnostic assessments. While direct evidence linking variations in WBA to different ethnicities is limited, research demonstrates racial variations in craniofacial and velopharyngeal morphologies in children, which could potentially affect sound energy transmission in the middle ear [20]. Developing normative data tailored to specific ethnicities is crucial for accurate and reliable diagnostic outcomes in pediatric audiology where WBA demonstrates unique characteristics (Figure 1c).

## 3. Characteristics of WAI in Ear Diseases

**Figure 2:**
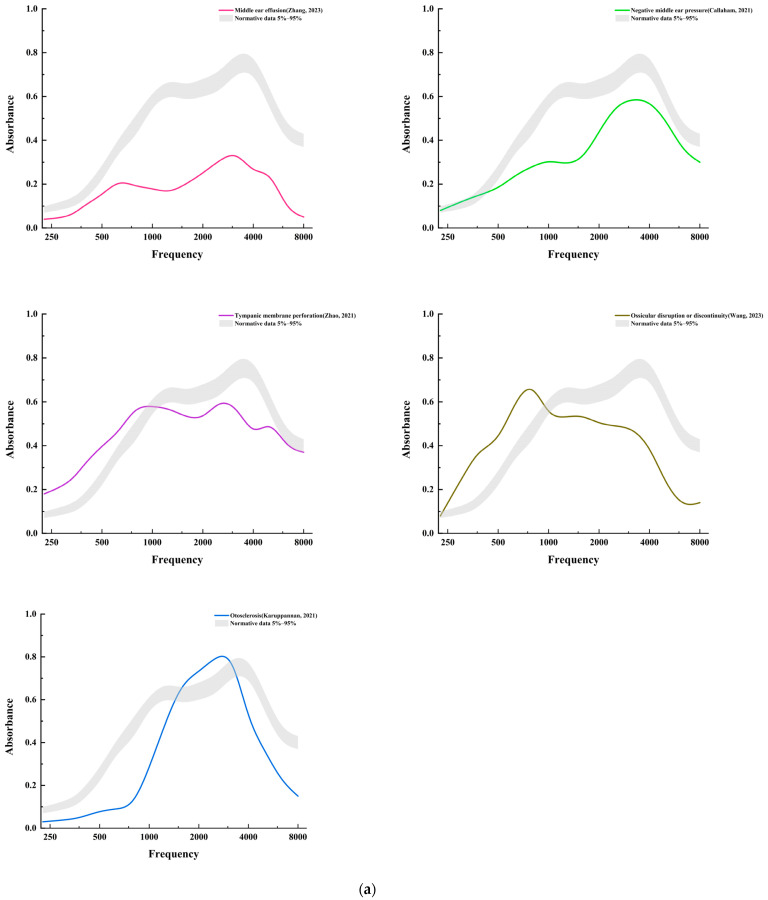

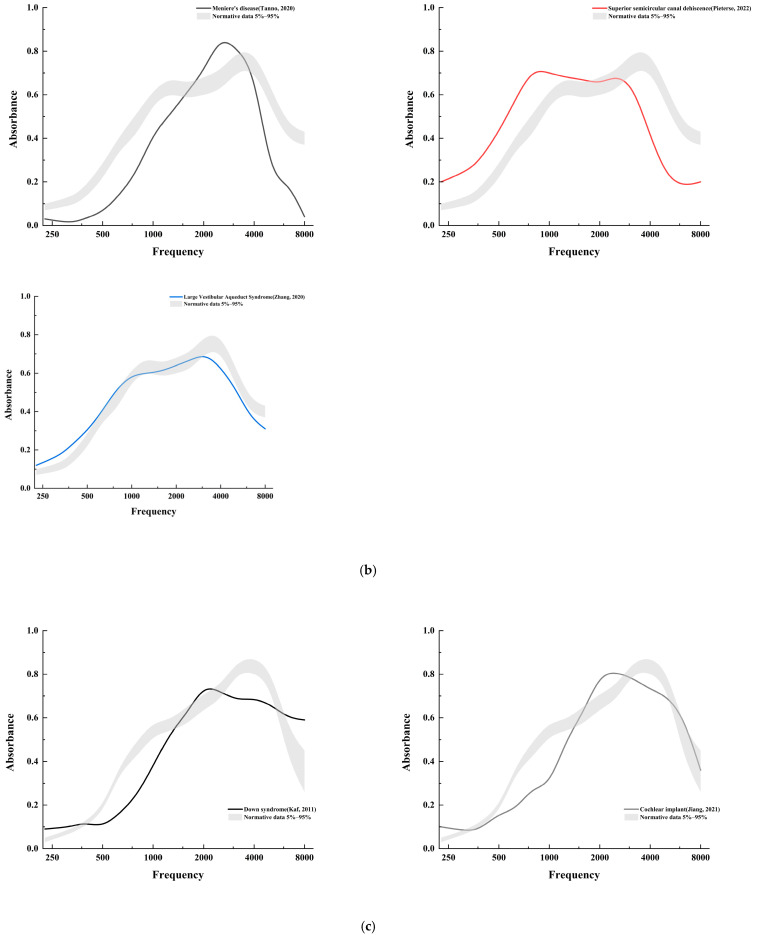
Analysis of the characteristics of (**a**) different middle-ear diseases [25,27,32,33], (**b**) different inner-ear diseases [2,29,30], and (**c**) other conditions [34,35]. Normative data: 5–95% [36].

The middle ear exhibits specific absorbance characteristics at different frequencies, lending significant clinical relevance to WAI in assessing middle-ear functional status. WBA demonstrates unique characteristic curves in different middle-ear diseases, diverging from those in children without middle-ear diseases [25,26,27,28]. This variance illustrates that WAI is exceptionally effective in discerning the etiology of conductive hearing loss, such as otitis media [25], otosclerosis [26], or ossicular chain malformations [27] (Figure 2a). Recent studies further affirm the high accuracy and predictive value of WAI in assessing pediatric conductive hearing loss [28].

Advancements have also been made in applying WAI to the study of inner-ear diseases, such as superior semicircular canal dehiscence [29] or large vestibular aqueduct syndrome [30] (Figure 2b). In these conditions, WBA often shows either increased or decreased absorbance at characteristic frequency bands, providing valuable diagnostic information. The concept of using WAI for diagnosing inner-ear diseases is linked to the energy transformation functions of the middle and inner ears. Some scholars have proposed a theory of stapedial footplate movement restriction [31]. This theory suggests that increased inner-ear pressure, such as from an enlarged vestibular aqueduct, pushes the stapedial footplate from the inside, limiting its movement. A probe placed in the external auditory canal can sensitively record these minute changes in the acoustic transmission pathway.

### 3.1. Middle-Ear Diseases

WAI is an effective tool for identifying reduced WBA in specific frequency ranges, offering an approach to diagnosing and understanding pediatric middle-ear diseases [3]. Otitis media with effusion (OME) is a common non-suppurative middle-ear inflammation in children. Some research by Shahnaz et al. [17] revealed that patients with OME have lower WBA across all frequency ranges compared with normal control groups. Merchant et al. [37] investigated the WBA in OME children with varying levels of effusion, discovering that the WBA curve tends to flatten as the volume of effusion increases. Studies by Sheng et al. [38] on children of different ages with OME via WAI analysis showed significant variations in WBA at different frequencies. Similarly, Gao et al. [39] pointed out that children with OME exhibit variations in WBA under peak pressure conditions across different age groups.

### 3.2. Inner-Ear Diseases

WAI is increasingly recognized as a valuable tool in assessing inner-ear diseases, such as Meniere’s disease [2,40], large vestibular aqueduct syndrome (LVAS) [30], superior semicircular canal dehiscence [6] and inner-ear malformations [5], which can impact sound transmission and lead to hearing loss. WAI assesses changes in sound energy reflection and absorption, aiding in the diagnosis and differentiation of these conditions.

Compared with other inner-ear diseases, LVAS is more commonly associated with hearing loss in children. LVAS is a significant congenital anomaly in the inner ear, leading to early-onset sensorineural hearing loss in children [41,42]. LVAS accounts for approximately 13 to 15% of pediatric SNHL cases [43,44,45]. Recent studies have focused on the WBA characteristics in LVAS patients (Table 1).

Jiang et al. [46] found that the WBA in the LVAS group was significantly lower than those in the control group at middle frequencies (1259–2000 Hz) but higher at high frequencies (4000–6349 Hz). Zhang et al. [30] also found that in children with LVAS, the average WBA under ambient pressure conditions is significantly reduced at specific frequencies (1000, 1189, 1296, 2000, and 4000 Hz). However, their research also noted that above 4000 Hz, the WBA under both ambient and peak pressure conditions exceed normal ranges, while below 500 Hz, the WBA is higher compared with control groups. Li et al. [21] indicated a higher WBA at low–mid-frequencies (343–1124 Hz and 1943–2448 Hz) in the LVAS group compared with control groups, while it was lower at high frequencies (3886–6727 Hz). Ding et al. [47] observed that at low frequencies (226–1000 Hz), the WBA in the LVAS group significantly exceeded that in non-LVAS children.

Jiang and Zhang’s studies indicated a decrease in WBA at mid-frequencies and an increase at high frequencies, while Ding’s study indicated an increase in WBA at low frequencies, and Li’s study indicated an increase in WBA at low–mid-frequencies and a decrease at high frequencies. These discrepancies highlight the variability in findings across different studies. However, Jiang et al. [46] highlighted that age may play a role in affecting WBA measurements, with older age leading to higher WBA values at high frequencies. The variations in age distributions and sample sizes between studies, as well as differences in study powers at different frequencies, suggest that direct comparisons should be interpreted cautiously.

Collectively, these studies suggest that compared with control groups, the WBA patterns in LVAS patients are distinctive, especially in certain frequency ranges. These findings highlight the need for more in-depth research and diverse analysis to integrate these findings and provide guidance for clinical practice. By integrating advanced techniques with clinical data analysis, future research may enhance the reliability of WBA for LVAS diagnosis.

### 3.3. Other Applications

In conditions such as Down syndrome (DS), WAI is utilized to detect distinctive patterns of WBA that differ from non-DS controls, aiding in the evaluation and management of auditory disorders. For patients with Cochlear implants (CIs), WAI is used to monitor changes in WBA at specific frequencies post-implantation. This application of WAI helps in the evaluation and management of auditory function in these patients.

Patients with DS often have anomalies in their outer, middle, and inner ears, leading to a higher incidence of auditory disorders [33,34]. A study by Kaf [35] found that even in DS patients with normal middle-ear status, their WBA patterns differed from those of non-DS control groups (Figure 2c). Soares et al. [48] expanded this analysis by comparing WBA in DS patients across different middle-ear conditions and found that those with abnormal tympanograms had a lower WBA than those with normal tympanograms.

Cochlear implants are an effective treatment for severe–profound sensorineural hearing loss. This implantation surgery alters the biomechanical properties of the middle and inner ears, changes that can be evaluated using WAI. Some research by Merchant et al. [49] found that ears with CIs show a reduced WBA at low frequencies compared with ears with normal hearing. This observation was further confirmed by studies by Wu et al. [50] and Jiang et al. [34] (Figure 2c), who noted significant differences in WBA between implanted and non-implanted ears.

### 3.4. Application Summary

WAI has demonstrated high accuracy and application value in the diagnosis of middle- and inner-ear diseases. In Table 1, an overview of the research subjects and main findings of WAI in the diagnosis of middle- and inner-ear diseases is provided.

**Table 1:** Key findings of wideband acoustic immittance (WAI) testing in pediatric audiology.

Condition	Author(s)	Study Group (n, age)	Key Findings
Otitis media	Merchant et al. (2023) [51]	63 ears, 9 months to 11 years 2 months	The analog model produced good fits for all effusion volume (full, partial, or clear) groups, which can estimate behavioral audiometric thresholds within a margin of error that is small enough to be clinically meaningful.
	Merchant et al. (2021) [37]	49 ears, 9 months—11 years	A multivariate logistic regression approach was utilized. WBA is a strong and sensitive indicator of OME.
	Liang et al. (2021) [52]	136 ears, 3–7 years	WBA is an effective method of diagnosing OME in children. The frequency band with the most predictive value of WBA for OME is 0.47–1.03 kHz.
	Aithal et al. (2020) [53]	60 ears, 5.5 ± 3.3 years	WBA demonstrated a high test performance comparable to 226-Hz tympanometry.
	Zhang et al. (2023) [32]	56 ears, 5.82 ± 3.04 years vs. 78 ears, 6.56 ± 2.86 years vs. 70 ears, 5.97 ± 2.75 years	A negative correlation was found between the middle-ear resonance frequency and effusion viscosity, as well as the air-bone gap.
	Callaham et al. (2021) [33]	211 ears, mean age: 2.73 years	WBA can differentiate between types of middle-ear effusion (serous, mucoid, or purulent).
	Pan and Yang [15]	342 ears, 2–16 years	WBT’s utility in diagnosing OME was explored.
	Keefe et al. (2012) [28]	35 ears, 3.5–8.2 years	WBA is a more accurate predictor (97–99% accuracy) of OME compared with traditional 226 Hz tympanometry (80–93% accuracy).
	Beers et al. (2010) [16]	64 ears, mean age: 6.34 years	Ethnic differences were found in the energy reflectance and effectiveness of WBA in distinguishing normal ears from those with MEE.
Cochlear implant	Jiang et al. (2021) [34]	20 ears, 6–8 years	A significantly lower WBA was found in the OME group compared with the control group under different pressure conditions.
	Wu et al. (2021) [50]	12 ears, 6–8 years and 2.52 ± 0.51 years	The WBA characteristics in infants with cochlear implants were studied.
Down syndrome	Kaf (2011) [35]	19 ears, 2½–5 years	The WBR in children with Down syndrome was analyzed, revealing unique patterns.
	Soares et al. (2016) [48]	42 ears, 2–16 years	WBR was investigated as a diagnostic tool in children with Down syndrome.
Inner-ear malformations	Kaya et al. (2020) [5]	107 ears, 3–37 years	The WBA in various inner-ear malformations was examined.
Large vestibular aqueduct syndrome	Jiang et al. (2024) [46]	82 ears, 6 months–11 years	Lower WBA values at 1259–2000 Hz and higher values at 4000–6349 Hz were found.
	Li et al. (2023) [21]	38 ears, mean age: 57 months	A higher WBA at low–mid-frequencies (343–1124 Hz and 1943–2448 Hz) was found in the LVAS group compared with the control groups, while it was lower at high frequencies (3886–6727 Hz).
	Ding et al. (2021) [47]	40 ears, 3–11 years	A higher WBA at 226–1000 Hz was found.
	Zhang et al. (2020) [30]	24 ears, 3–9 years	A lower WBA at 1000, 1189, 1296, 2000, and 4000 Hz was found.

## 4. Further Clinical Implications with Case Studies

WAI has emerged as a valuable tool in the diagnosis and management of both middle-ear and inner-ear diseases. Its capability to provide detailed, frequency-specific information about middle-ear mechanics is particularly advantageous in pediatric audiology, where traditional diagnostic methods may be inadequate. The preceding sections have highlighted various applications of WAI, demonstrating its effectiveness in diagnosing a range of auditory dysfunctions.

To further illustrate the practical applications and benefits of WAI, we present two case studies: one involving LVAS and the other focusing on cholesteatoma. These case studies provide concrete examples of how WAI can be integrated into clinical practice to enhance diagnostic accuracy and improve patient outcomes. The case studies are derived from clinical observations and data collected at our institution, ensuring that they are relevant and representative of real-world clinical scenarios.

## 5. Case 1: Bilateral Large Vestibular Aqueduct Syndrome (LVAS)

A 1-year-old female child presented with bilateral LVAS, an example that further emphasizes the clinical utility of WAI in pediatric audiology.

### 5.1. Patient Background and Initial Assessment

**Figure 3:**
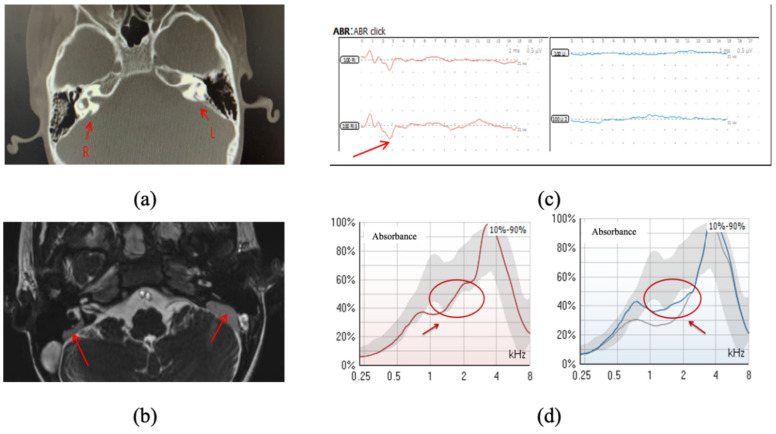
(**a**) CT findings (the red arrows indicate the enlarged vestibular aqueducts in right (R) and left (L) ears); (**b**) MRI findings (the red arrows point to the expanded endolymphatic sacs in both ears); (**c**) auditory brainstem response with the results of no response at 100 dBnHL in both ears. The red arrows mark the acoustically evoked short-latency negative response (ASNR), a large, negative deflection with a latency of approximately 3 milliseconds, which has been reported in patients with profound hearing loss [54,55]. ASNR is believed to originate from the saccule [55]. This response indicates the earliest neural activity in response to acoustic stimuli and is used to assess the integrity of the auditory pathway when typical ABR waves are absent [54,55]; (**d**) frequency–absorbance curve under the peak and ambient pressure conditions (the red circles, indicated by the red arrows, mark the frequency range that is significantly lower than the normal values).

The patient, diagnosed with bilateral LVAS with CT and an MRI scan, exhibited significant hearing impairment. The CT and MRI scans revealed bilateral enlargement of the vestibular aqueducts accompanied by expansion of the endolymphatic sacs (Figure 3a,b). Auditory brainstem response (ABR) indicated no response at 100 dBnHL in both ears, and acoustically evoked short-latency negative responses (ASNR) were shown in the right ear (Figure 3c). Tympanometry at 226 Hz indicated type A tympanograms for both ears, suggesting normal middle-ear function. 

### 5.2. WAI Findings

Both ears showed a significant decrease in wideband absorbance within the 1000 to 2519 Hz range. This specific pattern of reduction, or ‘notch’, at these frequencies is characteristically associated with LVAS (Figure 3d).

### 5.3. Clinical Significance

This case study illustrates the diagnostic capability of WAI. While the ABR and 226 Hz tympanometry offered basic information about the patient’s hearing status and middle-ear function, WAI provided a more detailed analysis of the ear mechanics, crucial for understanding the nature and extent of the acoustic transmission associated with LVAS.

The WAI findings for this case were instrumental in guiding clinical decision making. The identified notch in the WBA spectrum was critical in confirming the early diagnosis of LVAS, beyond the structural abnormalities detected through CT or MRI. Furthermore, the detailed WAI data assisted in a more effective management and treatment plan for the patient, considering the specific frequencies most affected by the condition. This information allowed clinicians to tailor interventions more precisely, such as focusing on amplification at the affected frequencies. Additionally, the WAI data provided valuable cross-validation with other audiological assessments, supporting the development of personalized treatment and rehabilitation plans. By understanding the exact nature of the frequency-specific impairments, treatment could be more accurately directed, improving overall patient outcomes.

## 6. Case 2: Cholesteatoma

A 13-year-old male child presented with cholesteatoma, an example that further emphasizes the clinical utility of WAI in pediatric audiology.

### 6.1. Medical History

Approximately three years ago, the parents noticed a decline in his hearing, accompanied by occasional tinnitus like a “ringing bell”. There were no reports of ear fullness, ear pain, headaches, dizziness, or other discomforts.

### 6.2. Clinical Examination

The child’s external auditory canals were found to be clear, with no cerumen or signs of inflammation. Both tympanic membranes appeared intact and displayed distinct landmarks. The mastoid region was palpated, revealing no tenderness or discomfort.

Audiological evaluation via pure-tone audiometry presented that the right ear fell within the normal hearing range. However, the left ear exhibited moderate conductive hearing loss, with an average air-conduction pure-tone threshold at 60 dB HL, while the bone-conduction pure-tone threshold remained within normal limits. This air–bone gap highlighted the conductive nature of the hearing loss in the left ear.

Tympanometry conducted at 226 Hz yielded type “A” tympanograms for both ears, suggesting normal middle-ear function, which was not consistent with the results of pure-tone audiometry.

The click-evoked auditory brainstem response (c-ABR) test revealed a significant inter-aural discrepancy. The left ear exhibited a markedly elevated threshold for wave detection at 80 dB nHL, pointing toward hearing loss. The right ear demonstrated a normal response threshold at 20 dB nHL, which was consistent with the results of pure-tone audiometry.

**Figure 4:**
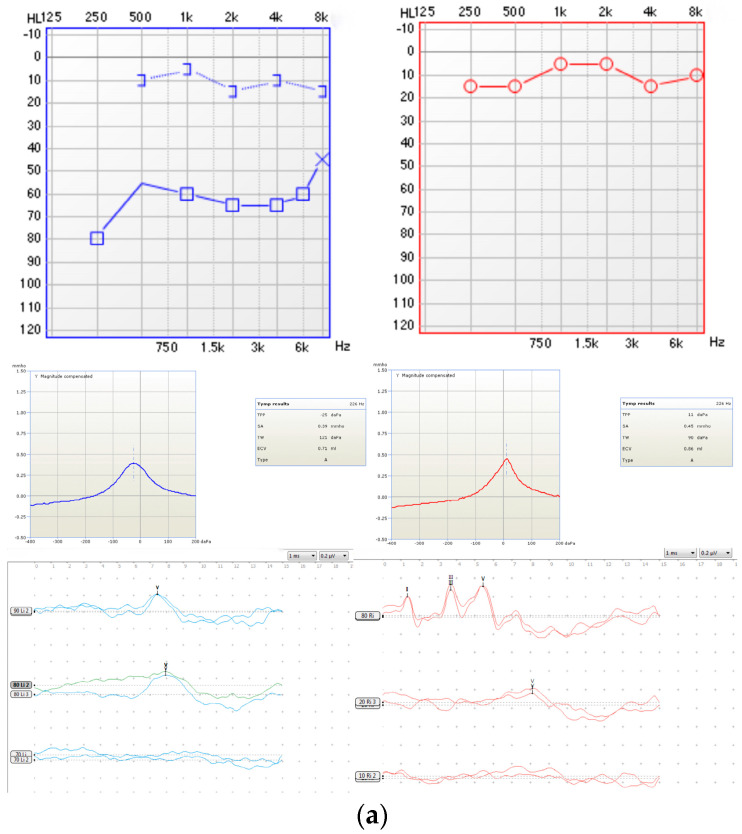

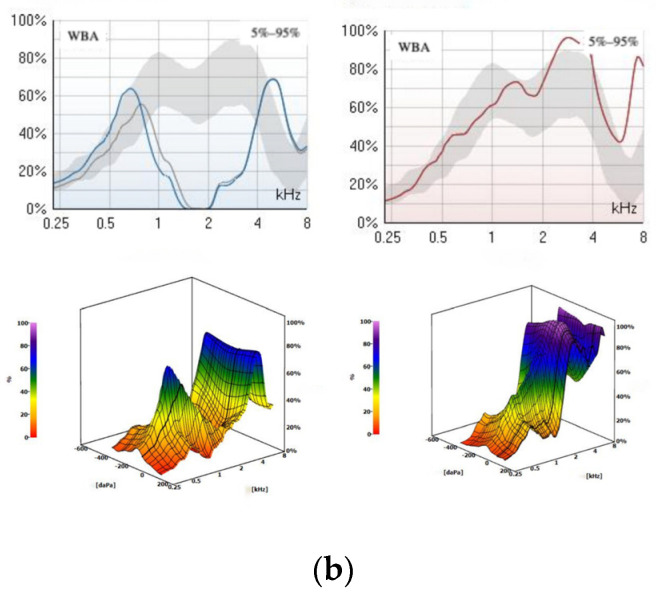
(**a**): Measurement results of pure-tone audiometry, 226 Hz tympanometry, and auditory brainstem response. (**b**): Two-dimensional and three-dimensional WAI graphical results. (The 2D representation of WAI data typically displays wideband absorbance as a function of frequency. The grey curve represents the WBA at ambient pressure, the blue curve presents the WBA at the tympanometric peak pressure (TPP) in the left ear, and the red curve presents the WBA at the TPP in the right ear. When the WBA is similar at ambient pressure and the TPP in the right ear, only the red curve is obvious. The 3D representation of the WAI data offers a more comprehensive view, integrating frequency, wideband absorbance, and pressure variations).

These clinical findings (Figure 4a), coupled with a confirmatory CT scan, led to the diagnosis of a cholesteatoma in the left ear. While ABR and PTA were pivotal in highlighting the discrepancy in audiological thresholds and prompting further investigation, WAI contributed additional diagnostic information by detecting subtle abnormalities in the middle-ear mechanics that were not evident in tympanometry. This non-invasive and quick assessment provided valuable insights that complemented the findings of ABR and PTA, reinforcing the need for a comprehensive audiological evaluation. Specifically, WAI detected frequency-specific anomalies in sound transmission, supporting the diagnosis of cholesteatoma. Thus, WAI adds a layer of diagnostic detail that enhances the overall assessment process, even if it does not replace the need for imaging studies like CT scans. This case underscores the critical role of comprehensive audiological assessment in identifying and characterizing ear pathologies, especially in instances where physical examination findings are unremarkable.

### 6.3. WAI Findings

The left ear demonstrated a significant decrease in the wideband absorbance at mid-frequencies, as shown in Figure 4b, indicating abnormal ear mechanics. In contrast, the right ear showed no abnormalities in the wideband absorbance. Cholesteatoma typically alters middle-ear mechanics by increasing mass and stiffness in the affected ear. This can result in reduced sound energy transmission and altered acoustic properties, as the cholesteatoma mass adds weight and disrupts the normal mobility of the ossicles and tympanic membrane. Since WAI is a new method for assessing middle-ear function, there is currently a lack of extensive research on its applications. Therefore, we cannot assert that cholesteatoma has a specific WAI pattern, which requires further scientific investigation. Moreover, conductive hearing loss with an intact tympanic membrane and normal middle-ear pressure typically necessitates additional investigation. However, WAI provides a quick, non-invasive, and frequency-specific assessment of middle-ear function, which can cross-validate the conductive hearing loss detected by pure-tone audiometry. This offers reliable audiological evidence for subsequent imaging examinations. The detailed absorbance data from WAI helps localize the dysfunction within the middle ear, suggesting the presence of an abnormal mass or increased stiffness, consistent with cholesteatoma. Consequently, the WAI results supported the decision to proceed with surgical exploration and tailored the intervention by highlighting specific areas of concern.

### 6.4. Surgical Intervention

During the exploration of the ossicular chain, normal mobility of the malleus was observed. However, the long process of the incus was found to be eroded, and a small cholesteatoma was present in the posterior tympanic cavity. The structures above the stapes footplate were destroyed, although the footplate itself was mobile. The incus was removed, and a titanium ossicular replacement prosthesis (TORP) was used to connect the stapes footplate and the handle of the malleus. The surgery aimed at reconstructing the ossicular chain, a critical procedure for restoring auditory function in cases of cholesteatoma where the ossicular chain is compromised.

### 6.5. Clinical Significance

This case of cholesteatoma in a pediatric child highlights the challenges in early diagnosis and the importance of timely surgical intervention. Cholesteatoma, a destructive and expanding growth in the middle ear, can lead to hearing loss and other complications if not treated promptly. The surgical approach in this case aimed at removing the diseased tissue and reconstructing the damaged ossicular chain, thereby improving hearing function.

This case underscores the complexity of managing cholesteatoma in children and the importance of a multidisciplinary approach that includes thorough clinical assessment, imaging, and appropriate surgical techniques for optimal patient outcomes.

## 7. Limitations

Despite its advancements, WAI’s application in pediatric audiology faces limitations, including variability in results due to different testing conditions. This information allowed clinicians to tailor interventions more precisely, such as focusing on amplification at the affected frequencies. Additionally, the WAI data provided valuable cross-validation with other audiological assessments, supporting the development of personalized treatment and rehabilitation plans. By understanding the exact nature of the frequency-specific impairments, treatment could be more accurately directed, improving overall patient outcomes. The technology’s effectiveness across diverse pediatric populations and various auditory conditions remains underexplored.

## 8. Future Studies

Future research should focus on standardizing WAI testing protocols and exploring its diagnostic accuracy across broader age ranges, ethnicities, and types of auditory disorders. Studies are needed to integrate WAI data with other diagnostic tools for a more holistic understanding of pediatric auditory pathologies.

## 9. Conclusions

This review recognizes wideband acoustic immittance as a significant advancement in pediatric audiology, offering comprehensive insights into various auditory disorders. However, it acknowledges the limitations due to testing variability and the necessity for further research to standardize protocols and explore their application across diverse age groups. Future studies are encouraged to enhance the diagnostic capabilities of WAI, ensuring a more accurate and holistic approach to pediatric audiology.

## Data Availability

The original data can be requested by emailing wen.jiang@zxhmu.edu.cn.
